# Upregulation of Small Ubiquitin-Like Modifier 2 and Protein SUMOylation as a Cardioprotective Mechanism Against Myocardial Ischemia-Reperfusion Injury

**DOI:** 10.3389/fphar.2021.731980

**Published:** 2021-09-13

**Authors:** Wei Zhao, Jia Zhao, Xiuying Zhang, Ni Fan, Jianhui Rong

**Affiliations:** ^1^ School of Chinese Medicine, Li Ka Shing Faculty of Medicine, The University of Hong Kong, Pokfulam, Hong Kong, SAR China; ^2^ Zhujiang Hospital, Southern Medical University, Guangzhou, China; ^3^ Shenzhen Institute of Research and Innovation, The University of Hong Kong, Shenzhen, China

**Keywords:** puerarin, myocardial ischemia/reperfusion injury, SUMO2, sumoylation, ERK

## Abstract

**Background:** Small ubiquitin-like modifier (SUMO) proteins modify proteins through SUMOylation as an essential protein post-translational modification (PTM) for regulating redox status, inflammation, and cardiac fibrosis in myocardial infarction. This study aimed to investigate whether natural product puerarin could alleviate myocardial ischemia/reperfusion injury (MI-RI) by targeting protein SUMOylation.

**Methods:** Mouse MI-RI model was induced by ligating the left anterior descending (LAD) coronary artery and subsequently treated with puerarin at the dose of 100 mg/kg. Rat cardiomyocyte H9c2 cells were challenged by hypoxia/reoxygenation and treated with puerarin at concentrations of 10, 20, and 40 μM. The infarction area of mouse hearts was assessed by 2% TTC staining. Cell damage was analyzed for the release of lactate dehydrogenase (LDH) in serum and cell culture medium. Western blot technique was employed to detect the expression of SUMO2, phospho-ERK, pro-inflammatory biomarker COX2, fibrosis index galectin-3, apoptosis-related protein cleaved PARP-1. The activation of the estrogen receptor (ER) pathway was assayed by the dual-luciferase reporter system.

**Results:** The present study validated that puerarin effectively reduced myocardial infarct size and LDH release in the mouse MI-RI model. In the cell culture system, puerarin effectively decreased the release of LDH and the protein level of COX2, galectin-3, and cleaved PARP-1. Mechanistic studies revealed that puerarin increased the expression of SUMO2, SUMOylation of proteins and the activation of ER/ERK pathway in cardiomyocytes. ER, ERK and SUMO2 inhibitors attenuated the cardioprotective effects of puerarin.

**Conclusion:** Puerarin may alleviate myocardial injury by promoting protein SUMOylation through ER/ERK/SUMO2-dependent mechanism.

## Introduction

Myocardial infarction (MI) represents a critical clinical manifestation of coronary heart disease and jeopardizes the health and well-being of the global population at a broader age range. The existing revascularization strategies only relieve the clinical symptoms of MI within a short period and appear to be less effective in preventing adverse cardiac remodeling (Wang et al., 2020a). Moreover, the current clinical treatments are highly invasive, so well-trained surgical personnel should be in place to execute the surgery with high cost and risk of surgical complications (Frieden and Foti, 2021). Thus, the effort is urgently needed to develop effective drugs for the functional recovery of post-MI hearts.

**GRAPHICAL ABSTRACT:**
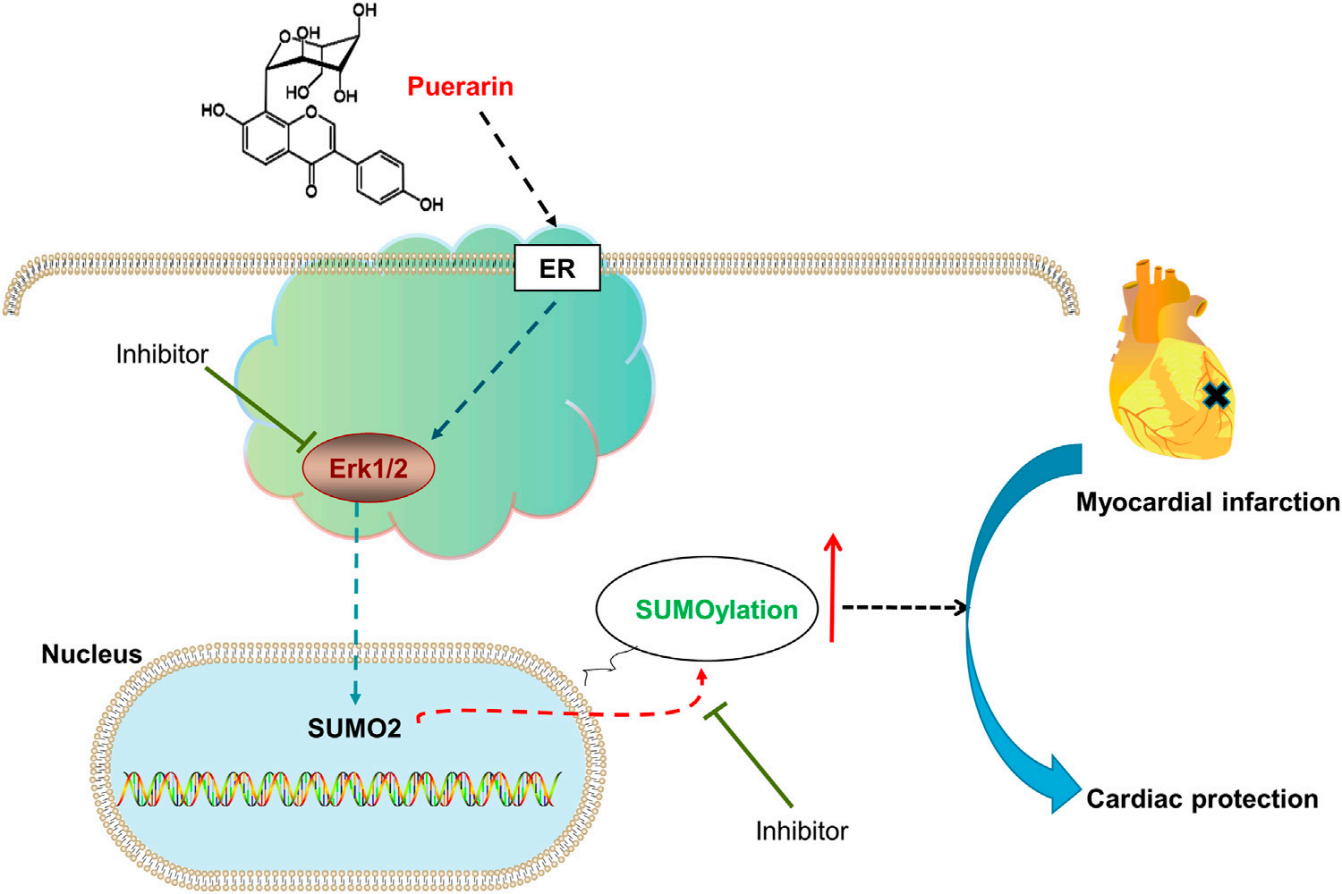


The pathology of ischemia and reperfusion is characterized by the dramatic increase of the cellular stress in the infarcted myocardium. Upon myocardial infarction, many proteins undergo different forms of post-translational modifications (PTM) and subsequently exhibit different or even opposite biological functions. It is well known that some proteins fail to function appropriately and others become hyperactivation in heart diseases due to the dysregulation of PTM, including phosphorylation, acetylation, glycosylation, amidation, hydroxylation, methylation, ubiquitylation and sulfation (Rookyard et al., 2021). These PTMs are dynamically balanced by conjugation and de-conjugation by functionally opposing enzymes (Li et al., 2021). Small ubiquitin-like modifier (SUMO) proteins conjugate with selected proteins by SUMOylation which is a new PTMs. SUMOylation and deSUMOylation are dynamically regulated for controlling the stability, cellular localization and activity of target proteins in the survival, proliferation, differentiation and apoptosis (Geiss-Friedlander and Melchior, 2007; Kho et al., 2015). It was recently found that SUMOylation could promote the adaptation of the heart to various pathological stress stimuli (Joung et al., 2018). SUMO overexpression enhanced cardiac functions in mice with heart failure and increased contractility in isolated cardiomyocytes (Kho et al., 2011). SUMOylation appeared to control the response of the heart to hypoxic/ischemic stress. Indeed, SUMO pathway components were dramatically changed during ischemia in the human heart (Sihag et al., 2009). These studies suggest that SUMOylation may be an important therapeutic target for drug discovery against myocardial infarction.

Herbal medicines are recently evaluated for targeting protein SUMOylation in animal models of MI and cell culture systems. Ginkgolic acid from the plant Ginkgo biloba might prevent cardiac fibrosis by inhibiting SUMO-1-dependent SUMOylation of proteins in MI (Qiu et al., 2018). Astragaloside IV from Radix Astragali stimulated angiogenesis under hypoxic conditions by enhancing SUMOylation of hypoxia-inducible factor-1α (HIF-1α) (Wang et al., 2021). Puerarin, 7,4-dihydroxyisoflavone -8β-C-glucopyranoside, is a major component of cardioprotective herbal medicine Radix Puerariae. Several recent studies suggested that puerarin could effectively protect the myocardium against ischemia and reperfusion injury (Li et al., 2019). Although the exact cardioprotective mechanisms are not well-defined, interestingly, our preliminary experiments showed that puerarin selectively up-regulated SUMO2 over SUMO1. Among four SUMO isoforms, SUMO2 is best known for its strong association with cellular stress. Therefore, the present study pursued two specific aims: 1) To explore whether puerarin could protect cardiomyocytes against myocardial infarction *via* up-regulating SUMO2 and related protein SUMOylation; 2) To discover the molecular mechanisms by which puerarin induced SUMO2 expression.

## Materials and Methods

### Antibody and Reagents

Puerarin was purchased from Yick-Vic Chemicals & Pharmaceuticals (Hong Kong, China). Fulvestrant, ML-792, and PD 98059 were purchased from Selleck Chemicals. Other biochemical reagents were purchased from Sigma-Aldrich (St. Louis, MO, United States) unless otherwise indicated. Dulbecco’s modified Eagle’s medium (DMEM), fetal bovine serum, penicillin, and streptomycin were purchased from Thermo Fisher Scientific (Waltham, MA, United States). Antibodies against COX2, ERK, p-ERK, galectin-3, and GAPDH were purchased from Cell Signaling Technology (Boston, MA, United States). Anti-8-OHdG was purchased from Santa Cruz Biotechnology Inc. (Dallas, Texas, United States). Anti-SUMO2 and Alexa Fluor 488-conjugated goat anti-mouse IgG secondary antibodies were purchased from Invitrogen (Carlsbad, CA, United States).

### Animals

All experimental procedures were approved by the Committee on the Use of Live Animals in Teaching and Research of the University of Hong Kong (CULATR 5636-21). Adult male C57BL/6N mice (8–12 weeks, 25–30 g) were supplied by the Centre for Comparative Medicine Research, University of Hong Kong, and housed in a humidity- and temperature-controlled environment on a 12 h light-dark cycle and allowed free access to standard laboratory mice chow and drinking water.

### Mouse Model of Myocardial Ischemia/Reperfusion Injury

To induce myocardial infarction, mice were anesthetized by i.p. injection of ketamine 100 mg/kg and xylazine 10 mg/kg under a mouse volume-control ventilator (55-7040, Harvard Apparatus, United States). Following thoracotomy between the 3rd and 4th intercostal space, surgery was performed to expose the heart and ligate the left main coronary artery with a 6–0 silk suture for 45 min. Following 45 min ischemia, the suture was loosened to allow reperfusion in the mice over 24 h for functional recovery. For drug treatment, puerarin was dissolved in 50% 1,2-propylene glycol in the saline. Puerarin 100 mg/kg was administered *via* i.p. injection at 30 min after ischemia, whereas a vehicle in equal volume was injected into the animals in Sham and I/R groups (Wenjun et al., 2015). After surgery, we monitored the animal’s consciousness and pain response in a well-conditioned environment. Fo the management of possible pain, mice were treated by i.p. injection of analgesic Buprenorphine (Temgesic®) at 0.1 mg/kg, 12-hourly for 3 days.

### Measurement of Myocardial Infarct Area Size

The mouse heart was harvested at the indicated time point, cut into five slices, and stained in 2% TTC for 15 min. The infarct area (IA) was characterized as a white region (Montaigne et al., 2018) and quantified by computerized planimetry of digital images using a free Downloadable NIH Image J software.

### Histopathological Examination (H/E Staining)

Histopathological examination was performed as previously described (Cheng et al., 2020). Briefly, when animals were fully euthanized, cardiac samples were collected from four groups, fixed in 4% paraformaldehyde in PBS and embedded in paraffin. After cutting into 5 slices, paraffin-embedded sections were stained with hematoxylin and eosin (H&E) stain and imaged under a light microscope. The images were assessed for gross myocyte injury and the effects of interventions.

### H9c2 Cells Culture and Hypoxia/Reoxygenation Model

Rat H9c2 cells were obtained from the American Type Culture Collection (Manassas, Virginia, United States) and cultured in DMEM (high glucose) containing 10% FBS, 100 U/mL penicillin, and 100 μg/ml streptomycin at 37°C in a humidified incubator containing 5% CO_2_. H9c2 cells were washed twice with PBS for the hypoxia challenge to remove glucose and serum and subsequently replaced with glucose-free DMEM with or without drug. The cells were exposed to the atmosphere of 0.1% O_2_ and 5% CO_2_ for 3 h in an Eppendorf Galaxy 48R hypoxia chamber (Hamburg, Germany). The cells were incubated in high-glucose DMEM containing 10% FBS for reoxygenation and placed in a 5% CO_2_ and 95% air incubator for 24 h (Li et al., 2020a). For drug treatment, the cells were grown to 70–80% confluence in the complete growth medium and treated with puerarin at the indicated concentrations for the specified times, whereas the control cells were treated with an equal amount of dimethyl sulfoxide (DMSO) under the same conditions. For Western blot analysis, the cells were pre-treated with puerarin for 1 h and subsequently stimulated with hypoxia and reoxygenation treatment for another 24 h.

### Assay of Lactate Dehydrogenase Release

The blood samples were collected from mice and centrifuged at 4,000 rpm at 4°C for 15 min for animal experiments. The serum was assayed for the level of LDH by the LDH Cytotoxicity Assay kit from Invitrogen (C20300, Waltham, MA, United States) following the manufacturer’s instruction. A cell culture medium was collected from injured H9c2 cells and assayed for LDH levels for the cell culture system. The absorbance at a wavelength of 490 nm was measured and quantified.

### Assay of Estrogen Receptor-Mediated Luciferase Activity

The activity of ER signaling pathway was assayed using a promoter-reporter system according to the manufacturer’s instruction. Briefly, H9c2 cells were seeded in 96-well plates at the density of 1 × 10^5^ cells/well, and transfected with 0.1 μg 3xERE-TK-Luc and 0.05 μg pRL-TK following the manufacturer’s instruction. After transfection for 24 h, puerarin and progesterone were used to treat the cells for another 24 h. ER transcriptional activity was then examined using the Dual-luciferase reporting system (Promega, United States) following the manufacturer’s protocol. The Clariostar microplate reader from BMG Labtech (Ortenberg, Germany) was used to detect luciferase activity.

### Western Blot Analysis

The expression of the cellular proteins was tested by Western blot analysis as described (Zhao et al., 2019). Briefly, heart tissues and H9c2 cells were lysed with RIPA buffer containing 1x protease inhibitor cocktail and centrifuged at 13,000 rpm for 20 min at 4°C. The supernatant was recovered, and a Bio-Rad protein staining reagent (Hercules, CA, United States) was used to determine the protein concentration. Proteins (60 μg for tissue samples, 30 μg for cells) were separated with 10% or 12% SDS-polyacrylamide gels and transferred onto polyvinylidene fluoride (PVDF) membranes (0.45 µm). The membranes were blocked through 2 h with 5% BSA. Then, the membranes were probed with primary antibodies overnight at 4°C and detected with HRP-conjugated secondary antibodies for another 1 h at room temperature. The blots were detected with Amersham ECT™ detection reagent (GE Healthcare, Uppsala, Sweden) following the manufacturer’s instruction. The fluorescence intensity was measured by Image J software (http://imagej.nih.gov).

### Cellular Immunofluorescence

H9c2 cells were treated with/without puerarin for 24 h, fixed in 4% paraformaldehyde, permeabilized with 0.5% Triton X-100 for 30 min, and blocked in 5% normal goat serum for 2 h at room temperature. Following incubation with anti-8-OHdG antibodies at 4°C overnight, the bound antibodies were detected with Alexa Fluor 488 anti-mouse IgG secondary antibody for 2 h at room temperature. After being washed with PBS three times, the cell nuclei were stained with DAPI for 5 min. The fluorescence microscopes (Carl Zeiss, Jena, Germany) were used to capture fluorescent pictures. The fluorescence intensity was measured by Image J software (http://imagej.nih.gov).

### Molecular Docking

The rat ER (PDB: 1X7R) structure was downloaded from the RCSB PDB website (http://www.rcsb.org/pdb). The chemical structure of puerarin was generated by ChemBioDraw Ultra 12.0 software. The protein-ligand interactions were simulated by the AutoDock Vina in the PyRx-virtual screen tool package (Trott and Olson, 2010). The docking results were analyzed by the Discovery Studio Visualizer software. The 3D and 2D models were generated using PyMol and LigPlus software, respectively.

### Statistical Analysis

The statistical analysis was performed by one- or two-way ANOVA (analysis of variance), followed by Dunnett’s test or LSD’s test using the GraphPad Prism software (GraphPad, CA, United States). The data were presented as mean ± SD from at least three independent experiments. The *p*-value of <0.05 was considered statistically significant.

## Results

### Puerarin Reduced Infarct Size and Cardiac Injury in Mouse MI-RI Model

**FIGURE 1:**
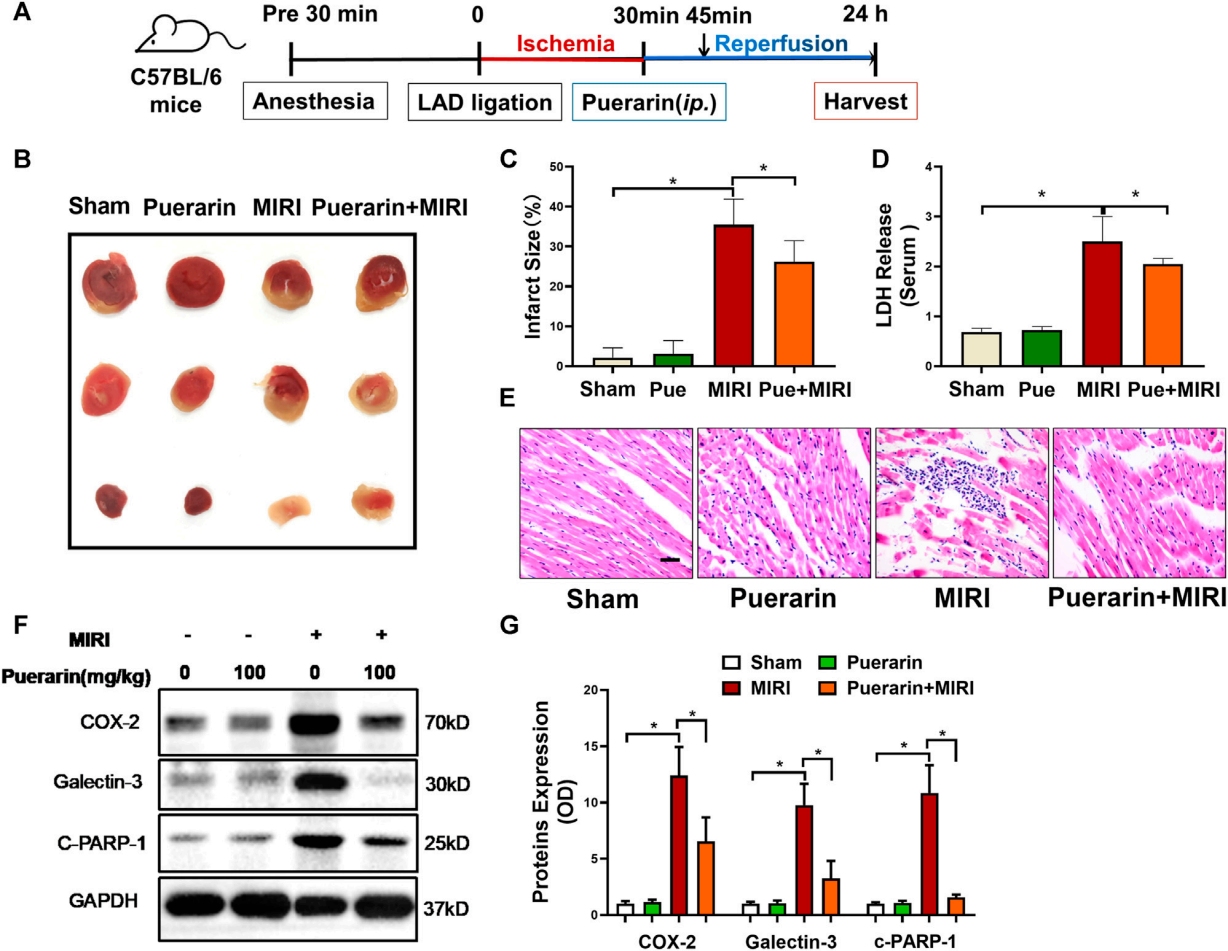
*In vivo* cardioprotective activity and anti-inflammatory effects of puerarin in mouse MI-RI model. **(A)** Outline of experimental design. C57BL/6N mice were subjected to LAD ligation to induce the MI-RI model. After 45 min of ischemia and 24 h of reperfusion, the hearts were harvested for further analysis. Puerarin 100 mg/kg was intraperitoneally injected 15 min before reperfusion. **(B)** Detection of myocardial infarction. After the tissue was stained in 2% TTC for 15 min, the infarct area (IA %) was assessed. **(C)** Quantitative analysis of infarct size. **(D)** Determination of serum LDH levels. The serum was collected from mice and determined for LDH levels. **(E)** H&E staining of myocardial tissue. Cardiac tissues were stained with H&E staining and imaged under light microscopy. Scale bar: 50 μm. **(F)** Western blot analysis of biomarkers. Cardiac tissues were collected and analyzed by Western blotting with antibodies against COX2, galectin-3, cleaved-PARP-1. **(G)** Quantification of COX2, galectin-3 and cleaved-PARP-1 expression. The results were shown as mean ± SD (*n* = 6). *p < 0.05.

The mouse model of MI-RI was induced by LAD ligation for 30 min and the mice were treated with or without puerarin 100 mg/kg for 24 h (Figure 1A). The myocardium was stained with 2% TTC while the infarct area (IA) was assessed. As shown in Figures 1B,C, puerarin effectively reduced infarct size (*p* < 0.05). Based on the determination of LDH content in the serum, puerarin significantly reduced LDH release from infarcted myocardium (Figure 1D; *p* < 0.05). H&E staining showed that MI-RI caused cardiac disorganization and massive infiltration of inflammatory cells, whereas puerarin effectively reversed MI-RI-induced damage (Figure 1E). Furthermore, pro-inflammatory protein COX2, fibrosis index galectin-3, apoptosis-related protein cleaved PARP-1 were up-regulated in infarcted cardiac tissue. Interestingly, puerarin largely reduced the upregulation of COX2, galectin-3, and cleaved PARP-1 in the mouse MI-RI model.

### Puerarin Protected H9c2 Cells Against Hypoxia/Reoxygenation Injury

**FIGURE 2:**
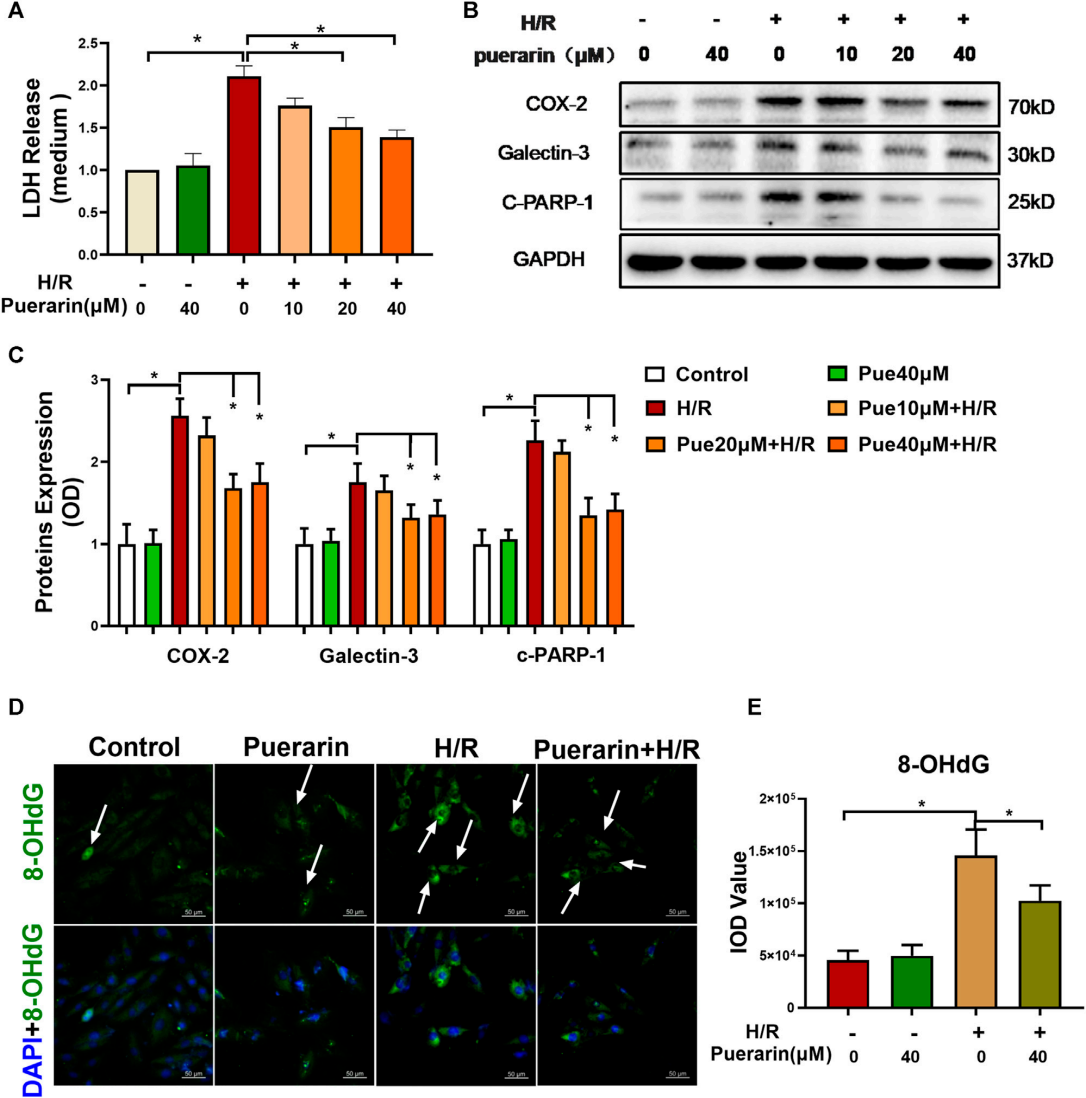
*In vitro* cardioprotective activity of puerarin against hypoxia/reoxygenation injury. **(A)** Determination of LDH release from H9c2 cells. After exposure to 3 h hypoxia (0.1% O_2_) and 24 h reoxygenation, H9c2 cells were treated with/without puerarin (10, 20, and 40 μM) for 1 h. LDH release was measured by a commercial LDH cytotoxicity assay kit. **(B)** Western blot analysis of COX2, galectin-3 and cleaved-PARP-1 expression. H9c2 cells were collected and the proteins were analyzed by Western blot with antibodies against COX2, galectin-3, cleaved-PARP-1. **(C)** Quantification of COX2, galectin-3 and cleaved-PARP-1 expression. The blots from Panel B were quantified by a densitometric method. **(D)** Immunostaining of 8-OHdG. H9c2 cells were probed with anti-8-OHdG antibody and visualized with Alexa Fluor 488-conjugated secondary antibody, whereas the cell nuclei were stained with DAPI. Scale bar, 50 μm. **(E)** Quantification of 8-OHdG generation. The results were presented as mean ± SD (*n* = 3). *p < 0.05.

To verify the *in vitro* cardioprotective effect of puerarin, H9c2 cells were challenged by sequential hypoxia (3 h) and reoxygenation (24 h). Firstly, the cell injury of H9c2 cells was evaluated by measuring LDH release from cells into the cell culture medium. As shown in Figure 2A, H9c2 cells released an enormous amount of LDH into the medium after hypoxia and reoxygenation, whereas puerarin reduced LDH release in a concentration-dependent manner (*p < 0.05). Secondly, Western blot analysis was used to examine the expression of COX2, galectin-3, and cleaved PARP-1 in H9c2 cells. As shown in Figures 2B,C, hypoxia and reoxygenation markedly up-regulated the expression of COX2, galectin-3, and cleaved PARP-1 (*p < 0.05), whereas puerarin (20 and 40 μM) reduced the levels of COX2, galectin-3 and cleaved PARP-1(*p < 0.05). Thirdly, 8-OHdG was measured as an indicator of DNA damage. As shown in Figures 2D,E, hypoxia and reoxygenation markedly increased the formation of 8-OHdG and the damage of the cellular DNA (*p < 0.05), whereas puerarin-40 μM effectively reduced the cellular levels of 8-OHdG and attenuated DNA damage (*p < 0.05).

### Puerarin Increased SUMO2 Expression and SUMOylation Levels in the Mouse Model and Cell Model of MI-RI

**FIGURE 3:**
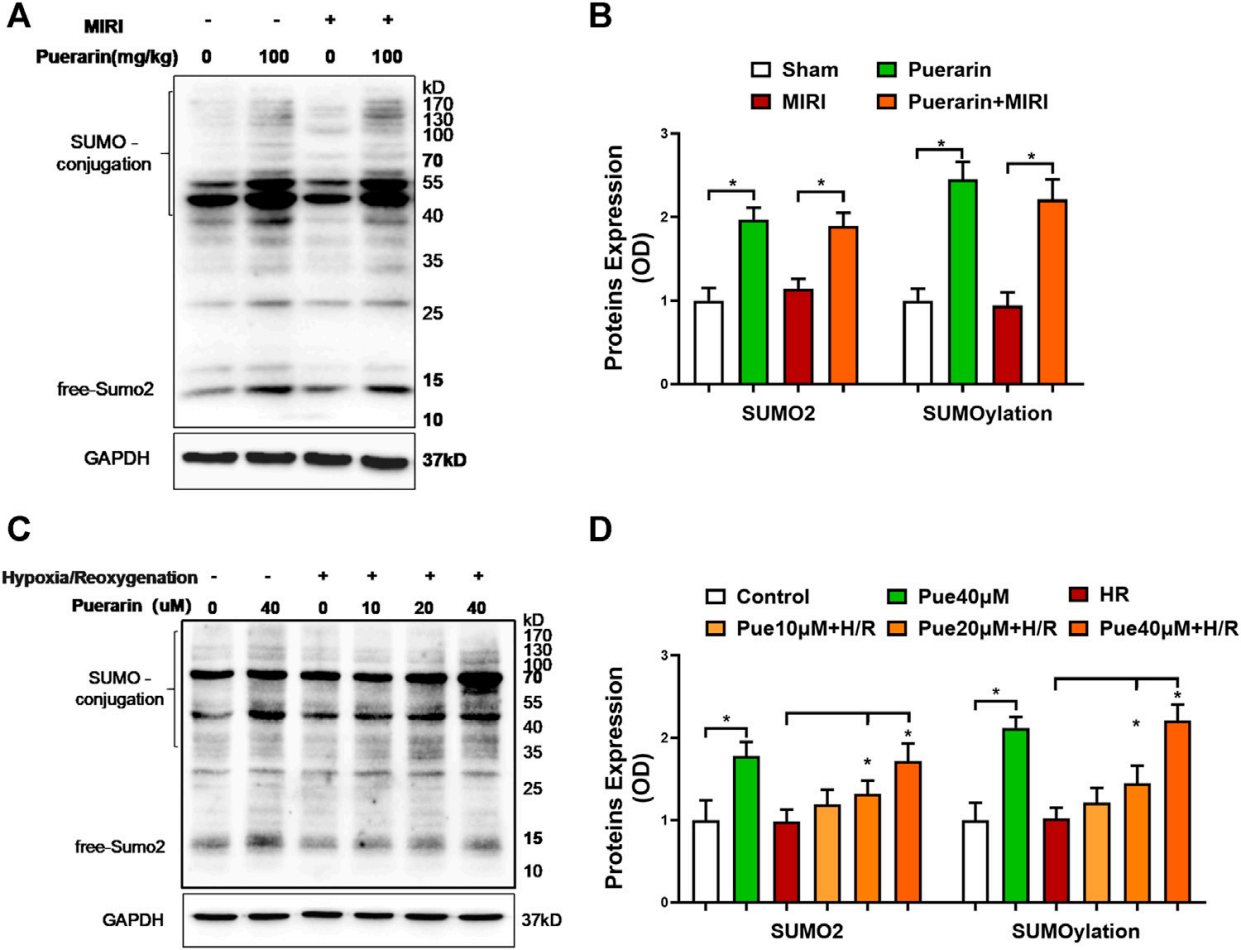
Effects of puerarin on SUMO2 expression and SUMOylation in mouse MI-RI model and hypoxia reoxygenation-challenged H9c2 cells. **(A)** *In vivo* effects of puerarin on SUMO2 expression and SUMOylation. After ischemia for 45 min and reperfusion for 24 h, heart tissues were collected and analyzed by Western blotting with antibodies against SUMO2, whereas GAPDH was analyzed as a control. **(B)** Quantification of SUMO2 expression and SUMOylation levels. The blots in panel A were quantified by a densitometric method. The results were shown as mean ± SD (*n* = 6). *p < 0.05. **(C)** *In vitro* effects of puerarin on SUMO2 expression and SUMOylation levels. After hypoxia (0.1% O_2_) for 3 h and reoxygenation for 24 h, H9c2 cells were collected and analyzed by Western blotting with antibodies against SUMO2, whereas GAPDH was analyzed as a control. **(D)** Quantification of SUMO2 expression and SUMOylation levels. The blots in panel C were quantified by a densitometric method. The results were presented as mean ± SD (*n* = 3). *p < 0.05.

To examine the effects of puerarin on SUMO2 expression and SUMOylation, we employed Western blot analysis to detect SUMO2 and SUMOylated proteins in mouse MI-RI model and hypoxia reoxygenation-challenged H9c2 cells. The free SUMO2 was detected to indicate SUMO2 expression, whereas the conjugated SUMO was detected to represent the levels of SUMO2-mediated SUMOylation. As shown in Figures 3A,B, puerarin 100 mg/kg effectively up-regulated SUMO2 and SUMOylation in both untreated mice and mouse MI-RI model (*p < 0.05). On the other hand, as shown in Figures 3C,D, puerarin could up-regulate SUMO2 expression and SUMOylation levels in hypoxia reoxygenation-challenged H9c2 cells in a concentration-dependent manner (*p < 0.05). Inconsistent with *in vivo* results, puerarin-40 μM alone increased SUMO2 expression and SUMOylation levels in untreated H9c2 cells.

### Puerarin Might Protect H9c2 Cells Against Hypoxia/Reoxygenation Challenge *via* Increasing SUMOylation

**FIGURE 4:**
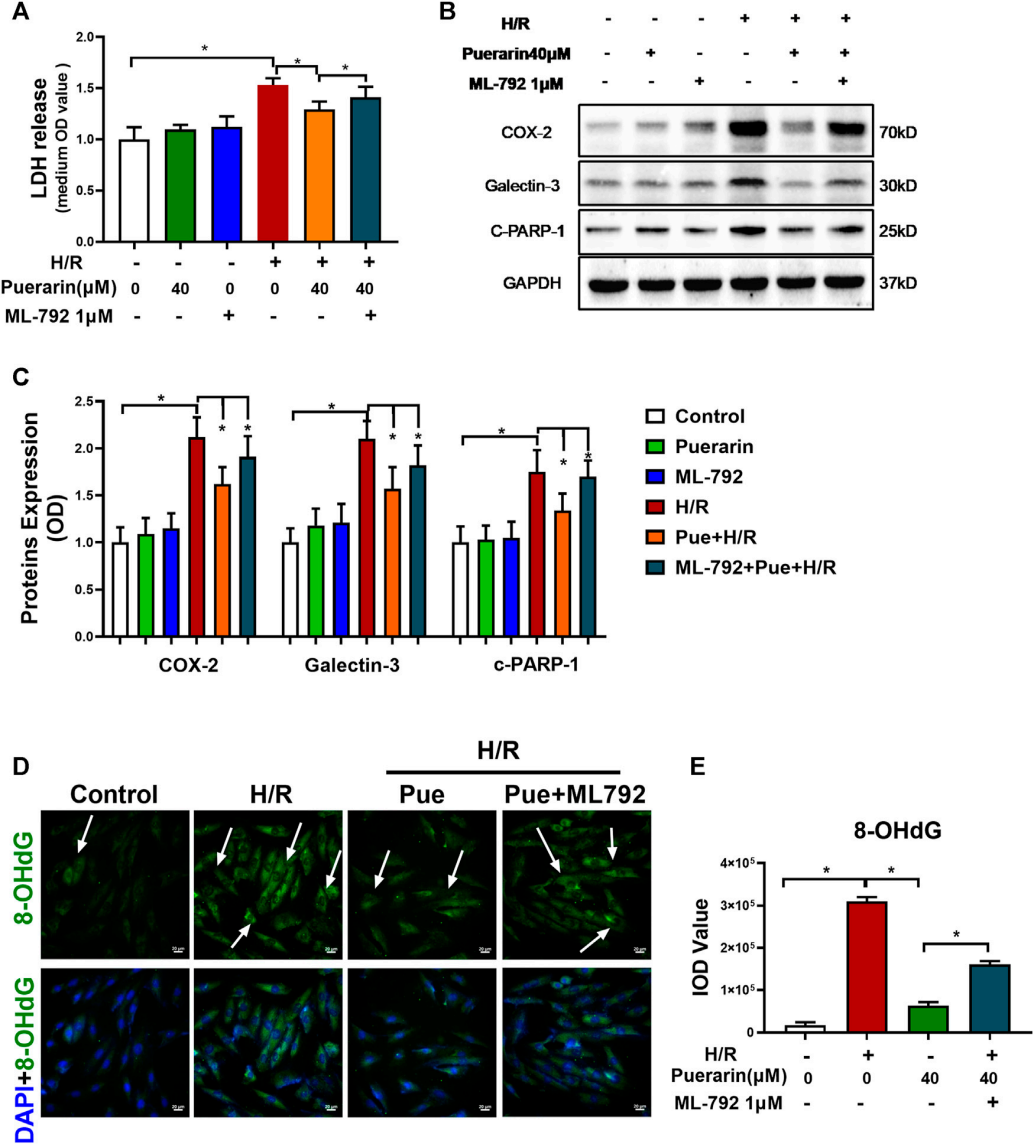
Puerarin promoted SUMOylation as a potential cytoprotective mechanism against hypoxia/reoxygenation challenge. **(A)** Detection of LDH release from H9c2 cells. After 1 h treatment with puerarin-40 μM and ML-792-1μM, alone or in combination, H9c2 cells were exposed to hypoxia (0.1% O_2_) for 3 h and reoxygenation for 24 h. LDH release was measured by the LDH cytotoxicity assay kit. **(B)** Western blot analysis for COX2, galectin-3 and cleaved-PARP-1 expression. H9c2 cells were collected and analyzed by Western blotting with antibodies against COX2, galectin-3, cleaved-PARP-1. **(C)** Quantification of COX2, galectin-3 and cleaved-PARP-1 expression. The blots in panel B were quantified by a densitometric method. The results were shown as mean ± SD (*n* = 3). *p < 0.05. **(D)** Immunostaining of cellular 8-OHdG. H9c2 cells were probed with primary anti-8-OHdG antibody and visualized with Alexa Fluor 488-conjugated secondary antibody, whereas the cell nuclei were stained with DAPI. Scale bar, 50 μm. **(E)** Quantification of 8-OHdG formation. The results were presented as mean ± SD (*n* = 3). *p < 0.05.

To clarify the role of SUMOylation in the *in vitro* cytoprotective effects of puerarin, before hypoxia/reoxygenation challenge, H9c2 cells were treated with puerarin alone or in combination with a specific SUMOylation inhibitor ML-792-1 μM. Firstly, the cell damage was assessed by assaying LDH release. As shown in Figure 4A, puerarin reduced LDH release from the cells that were exposed to hypoxia/reoxygenation challenge, whereas ML-792 significantly reversed the effect of puerarin on LDH release (*p < 0.05). Secondly, the Western blot technique was used to evaluate the effect of puerarin on the expression of COX2, galectin-3, and cleaved PARP-1 as the corresponding inflammatory, fibrotic and pro-apoptotic biomarkers. As shown in Figures 4B,C, puerarin significantly reduced the expression of COX2, galectin-3 and cleaved PARP-1 against hypoxia/reoxygenation challenge. ML-792 effectively reversed the effects of puerarin on the expression of COX2, galectin-3, and cleaved PARP-1 (*p < 0.05). Thirdly, the effect of puerarin on the DNA integrity was evaluated by immunostaining 8-OHdG. As shown in Figures 4D,E; puerarin effectively decreased 8-OHdG formation in the cells during hypoxia/reoxygenation challenge, whereas ML792 reversed the effect of puerarin on 8-OHdG formation (*p < 0.05).

### Puerarin Could Activate Estrogen Receptor in H9c2 Cells

**FIGURE 5:**
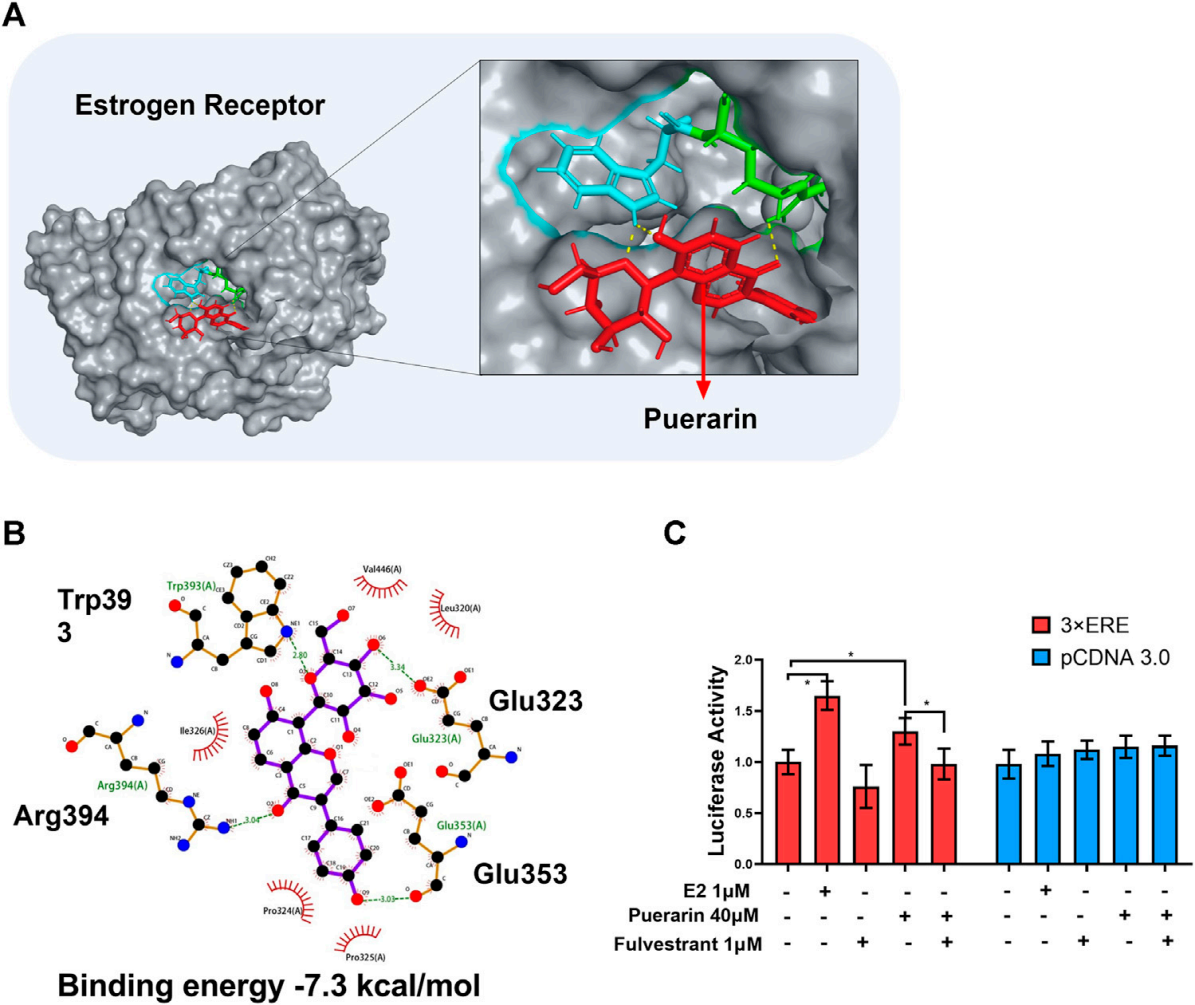
*In silico* binding of puerarin to ER and the activation of the ER pathway. **(A)** Molecular docking of puerarin to ER structure. Puerarin was docked to ER structure (PDB:1X7R) using software Autodock vina. **(B)** 2D plot illustrating the interactions of puerarin with amino acid residues of ER. Hydrogen bonds were shown in green lines. **(C)** Assay of ER-mediated gene transcription. Following transfection with 3xERE-TK-Luc and pRL-TK, H9c2 cells were treated with puerarin or estradiol and assayed for luciferase activity. The data were presented as mean ± SD (*n* = 3). *p < 0.05.

**FIGURE 6:**
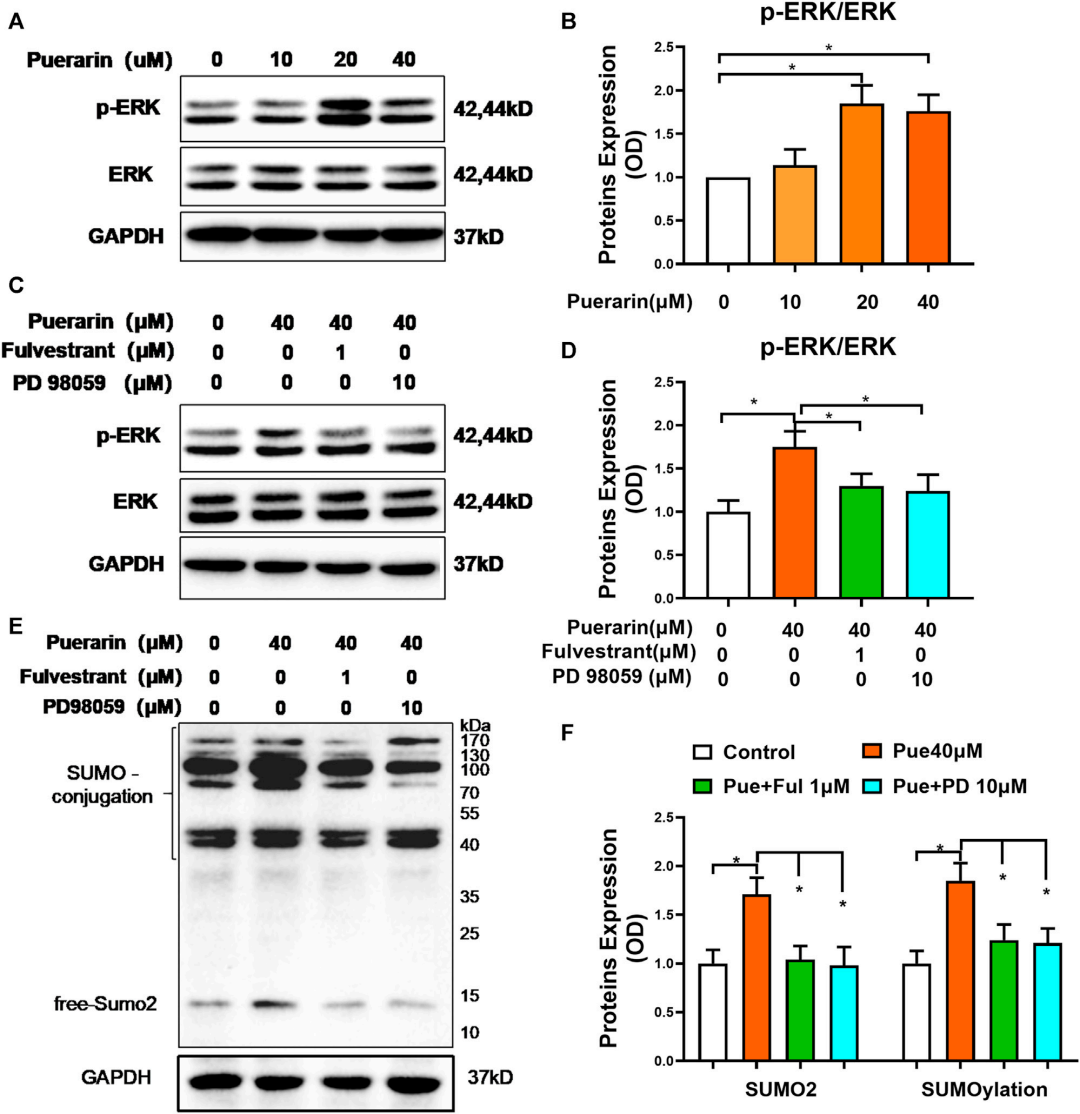
Puerarin might up-regulate SUMO2 and SUMOylation via the ER/ERK pathway. **(A)** Western blot analysis of p-ERK and ERK levels. Following treatment with puerarin (10, 20, and 40 μM) for 1 h, H9c2 cells were analyzed by Western blotting with antibodies against p-ERK and ERK. **(B)** Quantification of p-ERK and ERK expression. The results were shown as mean ± SD (*n* = 3). *p < 0.05. **(C)** Link of ER pathway to ERK activation. Following treatment with puerarin-40 μM alone or in combination with Fulvestrant-1 μM and PD 98059-10 μM for 1 h, H9c2 cells were analyzed by Western blotting with antibodies against p-ERK and ERK. **(D)** Quantification of p-ERK and ERK expression. The results were shown as mean ± SD (*n* = 3). *p < 0.05. **(E)** Western blot analysis for the role of ER pathway in the upregulation of SUMO2 expression and SUMOylation. Following the treatment with puerarin-40 μM alone or in combination with fulvestrant-1 μM or PD 98059-10 μM for 24 h, H9c2 cells were collected and analyzed by Western blotting with antibodies against SUMO2. **(F)** Quantification of SUMO2 expression and SUMOylation in panel E. The data were presented as mean ± SD (*n* = 3). *p < 0.05.

To explore the molecular mechanisms by which puerarin up-regulated SUMO2 expression, we focused on the ER signaling pathway. Firstly, puerarin was docked into ER crystal structure (PDB:1X7R). As shown in Figures 6A,B, puerarin is well bound to the site involving two amino acid residues (i.e., Trp393 and Arg394) in the ER structure while the binding energy was -7.3 kcal/mol. Secondly, the promoter-reporter system of the 3X ERE-TK-Luc plasmid construct was used to test whether puerarin could activate ER-mediated gene transcription in H9c2 cells. In practice, H9c2 cells were sequentially transfected with 3X ERE-Luc plasmid construct, treated with puerarin, and assayed for luciferase activity. As shown in Figure 5C, highly similar to the natural ligand E2, puerarin effectively increased luciferase activity *via* activating the ER pathway, while ER inhibitor fulvestrant reversed the effect of puerarin (*p < 0 0.05).

### Puerarin Promoted SUMO2 Expression and SUMOylation Through ER/ERK Signaling Pathway

To identify the involvement of the signaling molecules in the ER pathway, we detected the effects of puerarin on MAP kinases, including ERK in the H9c2 cells. As shown in Figures 6A,B, puerarin-20 μM effectively induced the phosphorylation of ERK in the H9c2 cells (*p < 0.05). As shown in Figures 6C,D, interestingly, ER inhibitor fulvestrant and ERK inhibitor PD 98059 suppressed the phosphorylation of ERK against the activity of puerarin (*p < 0.05). To clarify whether puerarin could up-regulate SUMO2 and SUMOylation *via* activating the ER/ERK pathway, H9c2 cells were treated with puerarin-40 μM alone or combined with Fulvestrant-1 μM and PD 98059–10 μM for 24 h. Based on Western blot analysis in Figures 6E,F, both Fulvestrant and PD 98059 inhibited the actions of puerarin on SUMO2 expression and SUMOylation (*p < 0.05).

### Puerarin Protected H9c2 Cells Against Hypoxia/Reoxygenation Injury *via* ER/ERK Pathway

**FIGURE 7:**
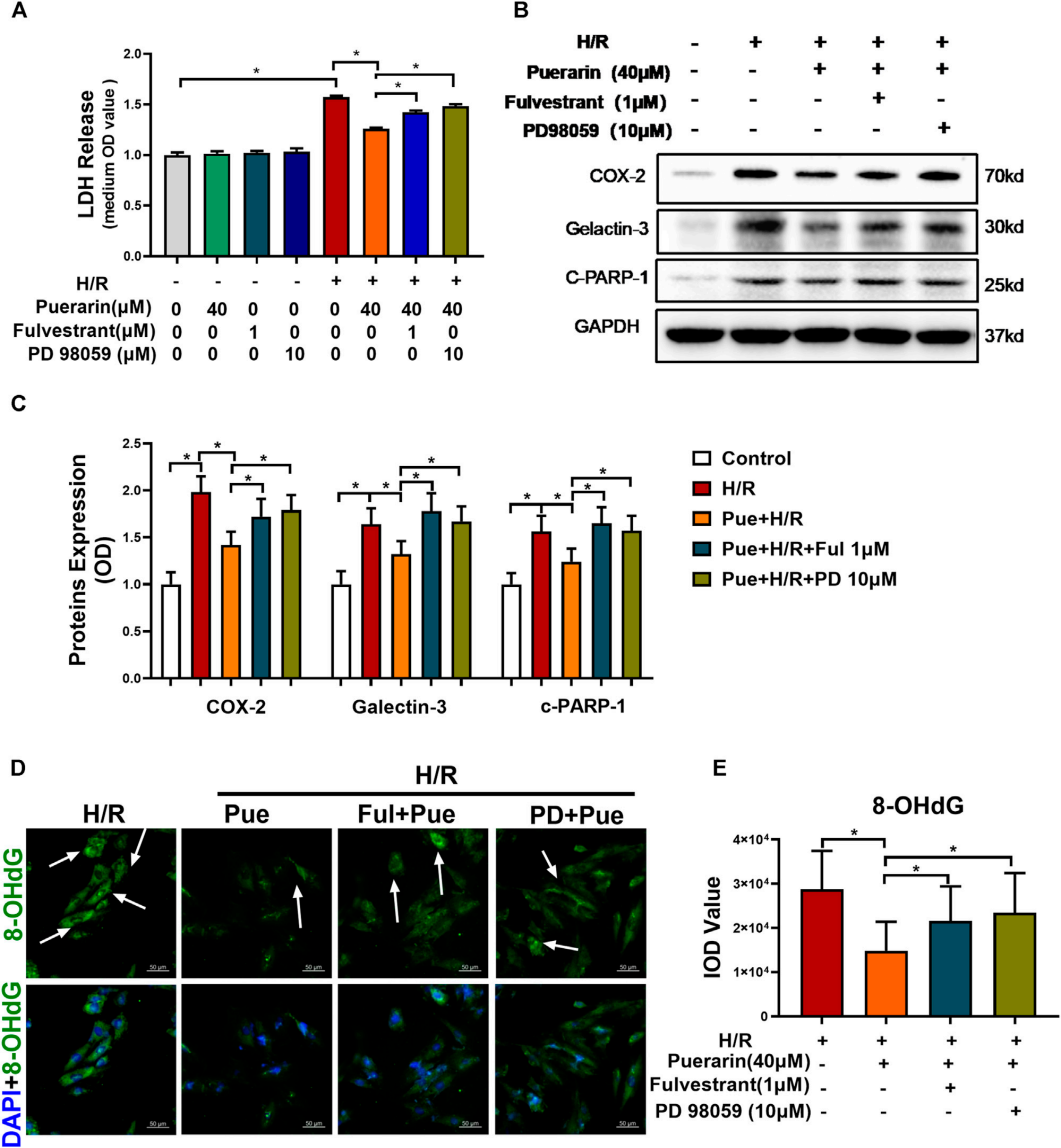
Puerarin protected H9c2 cells against hypoxia/reoxygenation injury via the ER/ERK signaling pathway. **(A)** Determination of LDH release. Following the treatment with puerarin-40 μM alone or in combination with Fulvestrant-1 μM or PD 98059-10 μM for 1 h, the H9c2 cells were challenged by hypoxia for 3 h and reoxygenation for 24 h and assayed for LDH release by LDH cytotoxicity assay (*n* = 3). **(B)** Western blot analysis of COX2, galectin-3 and cleaved-PARP-1 expression. Following drug treatment, H9c2 cells were collected and analyzed by Western blotting with antibodies against COX2, galectin-3, cleaved-PARP-1. **(C)** Quantification of COX2, galectin-3 and cleaved-PARP-1 expression. The results were shown as mean ± SD (*n* = 3). *p < 0.05. **(D)** Immunostaining of the cellular 8-OHdG. Following drug treatment, H9c2 cells were probed with primary anti-8-OHdG antibody and visualized with Alexa Fluor 488-conjugated secondary antibody, whereas the cell nuclei were stained with DAPI. Scale bar, 50 μm. **(E)** Quantification of 8-OHdG expression. The results were presented as mean ± SD (*n* = 3). *p < 0.05.

To further clarify the importance of the ER/ERK pathway in puerarin’s *in vitro* cardioprotective effects, H9c2 cardiomyocytes were treated with puerarin alone or in combination with Fulvestrant-1 μM or PD 98059-10 μM and subsequentially challenged by hypoxia/reoxygenation. Firstly, the cellular damage was examined by assaying LDH release. As shown in Figures 7A,B, puerarin reduced LDH release from H9c2 cells against hypoxia/reoxygenation challenge, whereas Fulvestrant and PD 98059 reversed the effects of puerarin on LDH release (*p < 0.05). Secondly, the effects of puerarin on cell survival, inflammation and fibrosis were examined by analyzing the expression of characteristic biomarkers (i.e., COX2, galectin-3, and cleaved PARP-1). As shown in Figures 7B,C, puerarin markedly reduced the expression of COX2, galectin-3, and cleaved PARP-1 in hypoxia reoxygenation-challenged H9c2 cells, whereas Fulvestrant and PD 98059 reversed the effects of puerarin (*p < 0.05). Thirdly, the effects of puerarin on DNA integrity were examined by detecting 8-OHdG formation. As shown in Figures 7D,E, puerarin decreased the cellular 8-OHdG level in hypoxia reoxygenation-challenged H9c2 cells, whereas Fulvestrant and PD 98059 reversed the effects of puerarin on hypoxia reoxygenation-induced DNA damage (*p < 0.05).

## Discussion

Therapeutic strategies are limited for resolving inflammation and preventing adverse cardiac remodeling in myocardial infarction (Toldo and Abbate, 2018; Zhao et al., 2020a). Different PTMs, including SUMOylation, are implicated in the pathological progression of myocardial infarction (Rookyard et al., 2021). The present study focused on the effects of natural product puerarin on SUMO2 expression and protein SUMOylation and the underlying mechanisms. We employed mouse MI-RI model and cardiomyocyte H9c2 cells to examine whether puerarin could protect cardiomyocytes against ischemia/reperfusion injury *via* up-regulating SUMO2 and SUMOylation. H9c2 cells were initially derived from embryonic rat ventricular tissue and could well model the responses of primary cardiomyocytes (Kimes and Brandt, 1976). Indeed, H9c2 cells are widely used as an important *in vitro* model of H/R injury (Ye et al., 2020; Chen et al., 2021). The present study also adopted H9c2 cells as the *in vitro* H/R model to examine the cardioprotective mechanisms.

Myocardial infarction triggers inflammatory responses, cardiomyocyte death, and cardiac fibrosis. Cardiac inflammation was hallmarked by the overproduction of pro-inflammatory biomarkers, including cyclooxygenase-2 (COX-2), an essential mediator of inflammation, toxic shock, and apoptosis (Guo et al., 2019; Zhang et al., 2021). Galectin-3 is a prognostic biomarker in heart failure and an essential mediator for cardiac fibrosis (Ho et al., 2012). Indeed, galectin-3 was markedly up-regulated in the myocardium and cardiomyocyte in response to ischemia/reperfusion and hypoxia/reoxygenation, respectively (Zhang et al., 2020; Redondo et al., 2021). Ischemia/hypoxia is well-known to cause cardiomyocyte death by activating caspase-3 and subsequent cleavage of PARP-1 protein (Zhao et al., 2018; Li et al., 2020b). Thus, COX2, galectin-3, and cleaved PARP-1 are often determined as the index for inflammatory response, apoptosis and fibrosis. Moreover, ischemia/reperfusion triggers enormous oxidative stress in the heart, causing aberrant oxidation of lipids, proteins, and DNA (Ucar et al., 2021). The cellular 8-OHdG is detected as a biomarker for oxidative DNA damage (Qin et al., 2007). This study assessed the *in vivo* and *in vitro* cardioprotective effects of natural product puerarin in mouse MI-RI model and H9c2 cells.

Puerarin is a major bioactive isoflavone from Chinese herbal medicine *Radix Puerariae*, well-documented for therapeutic effects against cardiovascular diseases, cerebrovascular diseases, neurological diseases and endocrinological diseases (Zhang et al., 2018; Zhou et al., 2021). Puerarin exhibits antioxidant activity for scavenging reactive oxygen radicals and prevents inflammation and apoptosis (Wenjun et al., 2015). Puerarin was also found to protect cardiomyocytes in myocardial infarction *via* regulating mitochondrial functions (Li et al., 2019). In the present study, the cardioprotective effects of puerarin were validated in the mouse MI-RI model as outlined in Figure 1A. The results shown in Figures 1B–G confirmed that puerarin could effectively reduce infarct size, inflammation, apoptosis and fibrosis in the heart after myocardial infarction. We were stimulated to explore the molecular mechanisms underlying the cardioprotective effects of puerarin. Thus, we challenged cardiomyocyte H9c2 by hypoxia/reoxygenation as the *in vitro* model for studying the underlying mechanisms. In practice, H9c2 cells were challenged by hypoxia and reoxygenation and subsequently treated with puerarin. The *in vitro* cardioprotective effects of puerarin were evaluated by measuring LDH release from the cells, analyzing COX2, galectin-3, and cleaved PARP-1 by Western blotting and detecting the cellular 8-OHdG by immunostaining. Indeed, the results in Figure 2 confirmed that puerarin could effectively exhibit cardioprotective effects in H9c2 cells against hypoxia/reoxygenation in a highly similar fashion. Such an H9c2-based *in vitro* model might support our effort to discover the mechanisms underlying the cardioprotective effects.

Protein SUMOylation involves different isoforms of SUMO proteins (Chang and Yeh, 2020). Even with high similarity at amino acid sequences, these SUMO isoforms may have overlapping functions and exhibit different functions. Previous studies suggest that SUMO2 may mediate the cellular response to stress (Mendler et al., 2016; Kim et al., 2019). Figure 3 revealed that puerarin could dramatically increase the expression of SUMO2 and the levels of SUMOylated proteins in mouse MI-RI model and H9c2 cells. We first addressed whether puerarin could protect cardiomyocytes against hypoxia/reoxygenation *via* up-regulating SUMO2 and protein SUMOylation. We examined whether specific SUMOylation inhibitor ML-792 could attenuate the effects of puerarin on LDH release. The expression of COX2, galectin-3, and cleaved PARP-1 and the formation of 8-OHdG. Figure 4 essentially demonstrated that the cardioprotective effects of puerarin were highly dependent on SUMO2-mediated SUMOylation. It was recently reported that over-expression of SUMO enhanced cardiac function in mice with heart failure and increased contractility in isolated cardiomyocytes (Kho et al., 2011). The present study further supported that SUMOylation might promote the adaptation of the heart to various pathological stress stimuli (Joung et al., 2018). Thus, SUMOylation may be a valuable target for the development of drugs against myocardial injury.

We subsequently examined how puerarin induced SUMO2 expression and protected cardiomyocytes against hypoxia/reoxygenation challenge. Like many other naturally occurring flavonoids, puerarin is classified as an estrogen receptor modulator and predominantly exhibits estrogenic activity for health benefits (Loutchanwoot et al., 2016; Hou et al., 2021). Figure 5 showed that puerarin was well bound to ER protein with the binding energy of −7.3 kcal/mol and induced the expression of reporter luciferase through an ER-mediated mechanism. Nevertheless, botanic drug puerarin may exhibit anti-inflammatory and cytoprotective activities through regulating different signaling pathways, including phosphoinositide 3-kinase (PI3K)/Akt pathway, NF-κB pathway and peroxisome proliferator-activated receptor (PPAR) pathway (He et al., 2019; Zeng et al., 2021). Our group previously found that puerarin might exhibit neuroprotective and neurorestorative activities *via* progesterone receptor (PR) signaling (Zhao et al., 2020b). These results confirmed that puerarin might interact with ER and PR in the cells.

For the role of ER downstream signaling molecules, it is well-known that the activation of ER signaling pathway results in the phosphorylation of the extracellular signal-regulated kinase (ERK) (Chen et al., 2019a; Zhao et al., 2021). Zhao et al. (2021) also found that puerarin activated Ras/mitogen-activated protein kinase/extracellular regulated kinase (MEK/ERK) and PI3K/Akt pathways *via* the non-genomic effects. Figure 6 showed that puerarin activated the ERK pathway through ER-mediated mechanisms. Both ER inhibitor Fulvestrant and ERK inhibitor PD 98059 attenuated the effects of puerarin on ERK activation, SUMO2 expression and SUMOylation in H9c2 cells. These results suggested that puerarin up-regulated SUMO2 expression and SUMO2-mediated SUMOylation in the ER/ERK-dependent manner. The SUMOylation affected the expression and function of proteins in myocardial infarction, which could attenuate or exacerbate myocardium injury through different mechanisms (Mendler et al., 2016; Bian et al., 2019; Chen et al., 2019b; Wang et al., 2020b). SUMOylation may partly enhance the level and functions of various proteins against myocardial injury (Chen et al., 2019b). Moreover, Figure 7 further supported that puerarin could protect H9c2 cells against hypoxia/reoxygenation insults *via* activating ER/ERK pathway. These results revealed that puerarin induced SUMO2 expression and enhanced protein SUMOylation and protected cardiomyocytes *via* activating ER/ERK pathway.

## Conclusion

In conclusion, the present study demonstrated that puerarin induced SUMO2 expression and enhanced SUMO2-mediated SUMOylation of proteins in mouse MI-RI model and H9c2 cells. Puerarin induced SUMO2 expression, enhanced SUMO2-mediated SUMOylation and protected cardiomyocytes *via* activating ER/ERK pathway. Importantly, SUMO2 and SUMO2-mediated SUMOylation are new important cardioprotective mechanisms. Thus, SUMO2-mediated SUMOylation may be a potential therapeutic target for the development of drugs against myocardial infarction.

## Data Availability

The original contributions presented in the study are included in the article/supplementary material, further inquiries can be directed to the corresponding author.
